# Study on the Semi-Solid Thixotropic Forging Forming Process for the Low-Carbon Steel Claw Pole

**DOI:** 10.3390/ma16134790

**Published:** 2023-07-03

**Authors:** Shuangjiang Li, Yongfei Wang, Zeyuan Li, Xiaoming Liu, Shengdun Zhao

**Affiliations:** 1 School of Mechanical Engineering, Xi’an Jiaotong University, Xi’an 710049, China; 2 State Key Laboratory of Compressor Technology (Anhui Laboratory of Compressor Technology), Hefei 230031, China; 3 Institute of Mechanical Manufacturing Technology, China Academy of Engineering Physics, Mianyang 621900, China

**Keywords:** semi-solid thixotropic forging, radial forging, isothermal heating, claw pole, microstructure

## Abstract

Low-carbon steel has been popularly applied in numerous applications because of its unique features, such as good plasticity, high strength, great hardness, and excellent toughness. Additionally, the semi-solid thixotropic forging forming method has been widely used in light alloys, due to its advantages of low forming force and high forming quality, whereas its application in ferrous materials is still limited. In this study, the semi-solid thixotropic forging forming process is proposed for producing the low-carbon steel claw pole, with the main stages being radial forging deformation, isothermal treatment, and forging forming. The effect of the area reduction rate on the effective strain from the cross sections of the radial-forged metal bar was studied using numerical simulations. The effect of the isothermal holding process on the microstructures of radial-forged billets was investigated, to obtain the ideal semi-solid microstructures. The microstructure and mechanical properties of low-carbon steel claw poles from the thixotropic forging experiment are presented and discussed. It was found that when the area reduction rate was 67%, the effective strain at the edge of the metal bar exceeded 5.0, while the effective strain at the center was above 1.2, indicating an excellent quality of forging for the bar. The optimization of the process parameters for preparing low-carbon steel semi-solid billets with fine and globular microstructures was achieved with an area reduction rate of 67%, an isothermal temperature of 1500 °C, and a duration time of 15 min. Moreover, the low-carbon steel claw pole fabricated with the optimized operating parameters was found fully filled, with a sharp profile and a flat surface, where the yield strength and tensile strength increased by 88.5% and 79.8%, respectively, compared to the starting materials.

## 1. Introduction

The demand for high-performance automobile parts is increasing with the rapid development of the automobile industry. The generator plays an important role in the automobile, and is usually applied to supply power to the other working components of the automobile [1]. As an important component of automobile generators, there is an ever-greater requirement for high dimensional precision and performance in claw poles. AISI 1010 low-carbon steel has been popularly applied in automobile generators, due to its excellent magnetic flux density, and AISI 1010 low-carbon steel rods consistently show the highest magnetic flux density values, compared to the AISI 1018, AISI 1045, and AISI 1045-high Mn/stress-proof steel rods [2]. The current forming process of low-carbon steel automobile generator claw poles mainly adopts multi-pass hot die forging, which has a large forming force, and a high requirement on the die [3,4]. Moreover, the traditional multi-pass hot die forging process of claw poles is long, the energy consumption is high, and there are defects, such as segregation, internal cracks, and pores in the formed parts. Therefore, it is very important to develop an advanced manufacturing process for low-carbon steel claw poles for automobile generators.

The semi-solid-metal-forming (SSMF) process [5,6] proposed by D.B. Spencer and M.C. Flemings, is an advanced forming process [7,8]. Compared with the hot die forging process, the forming force for the SSMF process is significantly decreased, due to the low flow stress and the good filling characteristic of the semi-solid metals, and the mechanical properties of the formed parts are close to those of the materials under the hot die forging condition [9,10,11]. As one of the SSFM processes, the semi-solid thixotropic forging forming (SSTFF) process, including the preparation of ideal semi-solid billets and the forging forming of components, has received extensive attention in recent years. During the SSTFF process, the semi-solid billets can be filled into the preheated die steadily under a small forging load, because the semi-solid billets are a mixed structure with fine, spherical solid grains and small surrounding liquid phases [12]. Moreover, the SSTFF process has a higher solid phase ratio and lower forming temperature than the rheocasting method, which can significantly reduce the thermal impact of the die, improve the service life of the die, and reduce the oxidation degree of materials in the forming process [13,14,15,16].

In recent decades, a large number of non-ferrous metal parts have been manufactured using the SSMF process for the application fields of the automobile industry, aviation industry, and electronics industry [17,18,19,20]. Kazemi et al. [21] employed the SSTFF process to form the A356 aluminum alloy centrifugal pump flange, and they found that the best mechanical properties of the centrifugal pump flange were obtained at the semi-solid temperature of 600 °C, holding time of 5 min, and ram speed of 5 mm/s. Kolahdooz et al. [22] discussed the effect of these forged parameters on the microstructures and mechanical properties of the helical gearbox cap, and the harness and forming load were decreased with the increase in the die temperature, billet temperature, and holding time. Cao et al. [15] investigated the forging process of the C3771 lead brass valve, and they reported that the cap thread and nut thread failure torque of the formed valve were also discovered to be higher than the traditionally forged copper valve with dendrite micro-grains. Chen et al. [23] studied the effects of the reheating temperature and time on the microstructure and tensile properties of this forged AZ63 magnesium alloy, and they found that the ultimate tensile strength of the resulting alloy was up to 310 MPa, with the billet temperature of 595 °C, the reheating time of 60 min, and the mold temperature of 350 °C.

Low-carbon steel has the properties of a high melting point, easy oxidation, and a narrow semi-solid temperature range. These properties not only increase the difficulty of the application of the semi-solid forming method, but also put forward challenges in the quality requirements for the manufacturing of the forming die [24,25,26]. Sugiyama et al. [24] have studied the semi-solid extrusion properties of low-carbon steel, and the microstructure of hot rolled bars obtained at semi-solid temperatures was similar to the spherical structure of aluminum and magnesium alloys. Becker et al. [25] studied the induction heating process of SAE1006 low-carbon steel to form tubes, achieving one-step forming with good dimensional and geometric tolerances. Aba-Perea et al. [26]. studied the thixotropic forming of low-carbon steel double-cup-shaped parts through a combination of numerical simulation and experiments, and found that compared to forging, the forming load decreased, and the extrusion length increased while maintaining mechanical properties. Therefore, compared with the traditional aluminum alloy and other light alloys [27,28,29], the application of the semi-solid forming method to low-carbon steel still has great room for expansion.

To the best knowledge of the authors, low-carbon steel claw poles formed using an SSTFF process including radial forging, isothermal treatment, and thixotropic forging forming have not been reported. Moreover, little research has been performed on the preparation of semi-solid AISI 1010 low-carbon steel spherical materials, and the forming quality of low-carbon steel claw poles. The SSTFF process is proposed in this work for producing the low-carbon steel automobile generator claw pole. The effect of the area reduction rate on the effective strain of the radial-forged metal bar, and the effect of the isothermal holding process on the macrostructures, the microstructures, and the mechanical performance of the claw pole obtained using the thixotropic forging forming process, were investigated through simulation and experiment.

## 2. Materials and Methods

### 2.1. Semi-Solid Thixotropic Forging Forming Scheme

**Figure 1:**
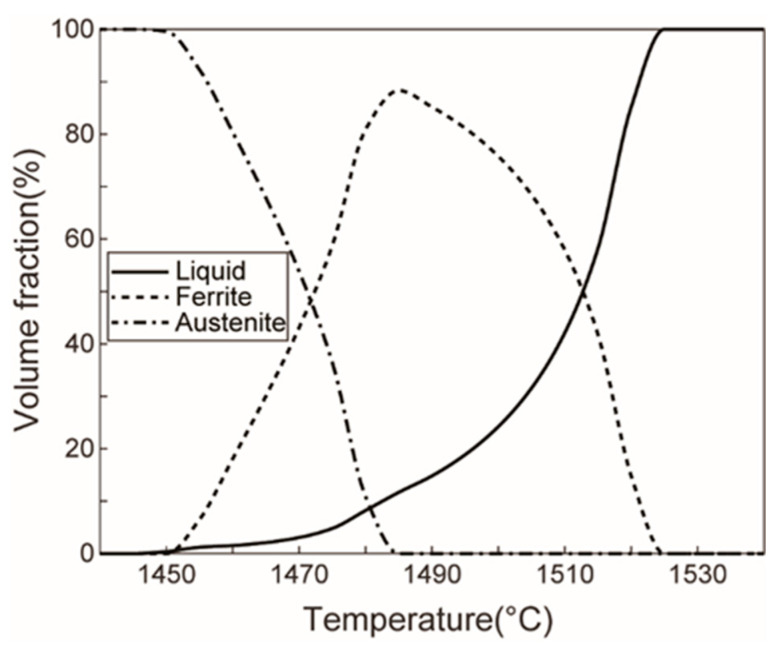
Temperature–phase curve of AISI 1010 steel.

**Figure 2:**
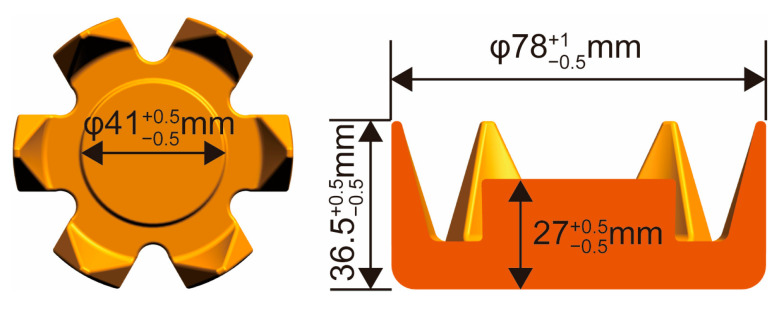
3D model and size of claw pole.

The material used in this study was annealed AISI 1010 low-carbon steel, and its chemical composition is shown in Table 1. The temperature-phase curve of AISI 1010 steel shown in Figure 1 was obtained from the JMatPro 7.0 software [30]. The chemical composition of AISI 1010 low-carbon steel, shown in Table 1, was inputted into the JMatPro 7.0 software, and then the temperature–phase curve of AISI 1010 steel could be automatically calculated. The volume fractions of each phase of AISI 1010 steel changed at different temperatures between the solidus and the liquidus. Near the solidus, the cementite in the low-carbon steel disappeared, and the main solid phase was austenite. With the increase in temperature, the austenite phase changed to the high-temperature ferrite component and the liquid phase. When the temperature rose to 1485 °C, all of the austenite phases were transformed into the high-temperature ferrite component and the liquid phase. The high-temperature ferrite component reached the maximum volume fraction of 88%, and the liquid-phase volume fraction was 12%. With the further increase in temperature to 1522 °C, the high-temperature ferrite component completely transformed into the liquid phase. Therefore, the solidus and liquidus temperatures of the studied material were 1450 °C and 1522 °C, respectively. Additionally, the information on the structure and dimensions of the claw pole is shown in Figure 2. It is a central symmetrical part, with six teeth all around the outer diameter, and the cross-section of each tooth decreases along the height.

**Figure 3:**
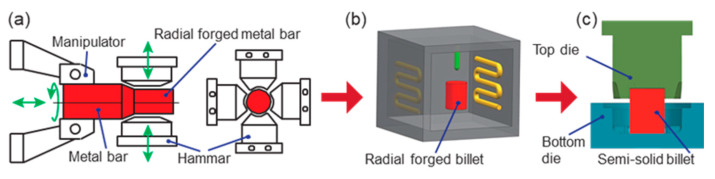
Process diagram of the semi-solid thixotropic forging forming method for the claw pole: (**a**) first stage—deformation, (**b**) second stage—isothermal treatment, and (**c**) third stage—forming.

The semi-solid thixotropic forging forming method of the low-carbon steel claw pole in this work was divided into three stages, as shown in Figure 3. In the first stage, the low-carbon bar was radial-forged with different section shrinkages at room temperature. In the second stage, the radial-forged materials were isothermal, and treated with different heating temperatures and holding times. In the third stage, the semi-solid billets with ideal microstructures were thixotropic-forged to obtain the low-carbon steel claw pole by the top and bottom dies. The schematic diagram of the radial forging process of the metal bar is shown in Figure 3a; the metal bar was forged by the four hammerheads in the radial direction, and then the metal bar was rotated along the radial direction, and shifted along the axial direction by the manipulator.

### 2.2. Simulation and Experimental Settings of Process Parameters

**Figure 4:**
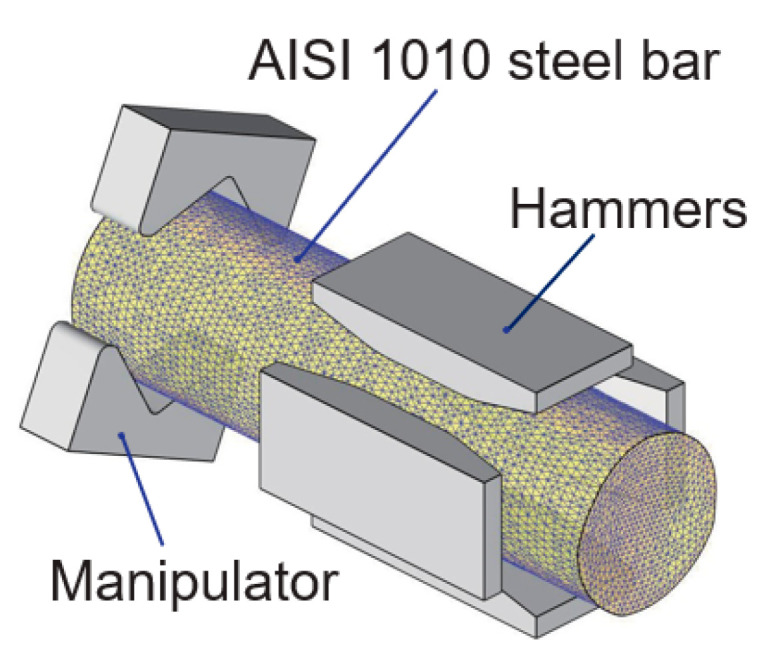
Three-dimensional structure of the radial forging simulation model.

The preparation of a semi-solid billet requires sufficient radial-forging plastic deformation of the material, before the isothermal heating procedure. The 3D simulation model of the radial forging method is shown in Figure 4, which mainly includes an AISI 1010 steel bar, a manipulator, and hammers. In the simulation, the stress-true strain curves of the AISI 1010 steel were obtained from the materials library of the DEFORM-3D 11.0 software. Moreover, the material of the hammers and manipulator in the simulation was set as H13 steel, while the heat transfer coefficient between the hammers and the AISI 1010 steel bar casting was set as 1500 W∙m^−2^∙K^−1^. Moreover, the following simplification and assumptions were made in this simulation:

(1) The hammers were regarded as a rigid body in the simulation calculation process.
(2) In the process of radial forging deformation, the elastic deformation of the billet is small, which has little influence on the deformation process. Therefore, the elastic deformation of the AISI 1010 steel bar was ignored, and only its plastic deformation was considered, and the AISI 1010 steel bar was regarded as a plastic body, and its own weight was ignored.

To ensure that the core of the low-carbon steel bar had sufficient plastic deformation, a steel bar with a diameter of 70 mm was divided into four radial forging steps. The final diameter of the low-carbon steel rod after radial forging was 40 mm, and the area reduction rate was 67%. The radial forging size scheme of the AISI 1010 steel bar in this study was designed as shown in Table 2. During the radial forging simulation process of the DEFORM-3D software, the equilibrium equations, constitutive relations, and boundary conditions were transformed into nonlinear equations by finite distance, and then the simulation was solved directly using the Newton–Raphson method [31,32].

**Figure 5:**
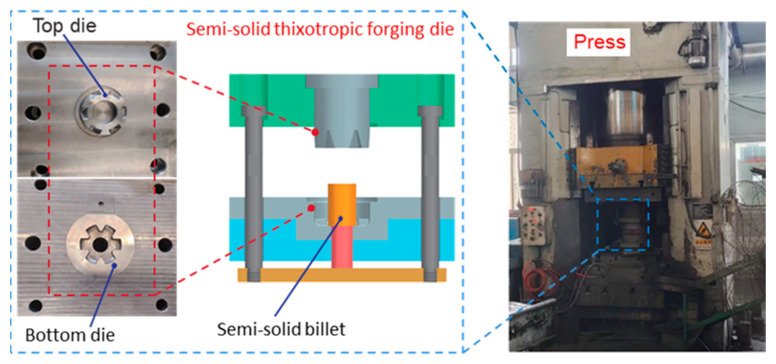
The semi-solid thixotropic forging dies and experimental press for the claw pole.

The experiment process for manufacturing the AISI 1010 steel claw pole was performed using the SSTFF process. Firstly, to obtain the ideal semi-solid materials for the SSTFF of the low-carbon steel claw pole, a resistance heating furnace was used to heat the radial-forged low-carbon steel bar. The preparation of semi-solid AISI 1010 steel materials was studied using four groups. In Group I, the heating temperature was set as 1490 °C, while the holding time was set as 10 min. In Group II, the heating temperature was 1490 °C, and the holding time was 15 min. In Group III and IV, the heating temperature was changed from 1490 to 1500 °C, and the holding time was set as 10 and 15 min, respectively. After this time, the average grain size was calculated and analyzed, to obtain the recommended process parameters for preparing semi-solid AISI 1010 steel materials with ideal microstructures. Secondly, the semi-solid billets with ideal microstructures were used for the thixotropic forging of the low-carbon steel claw pole. During the thixotropic forging process, the die preheat temperature was 300 °C, and the die closing speed was 45 mm/s. The semi-solid thixotropic forging dies and screw press are shown in Figure 5. As depicted in Figure 5, the semi-solid thixotropic forging die of the claw pole is composed of two parts, the bottom die is fixed, and the top die moves downward, to make the billets fill the die cavity.

The microstructures of the samples in the experiments were observed using an optical microscope, according to which the average diameter of the grains in the metallography structure was quantitatively obtained using Equation (1):

(1) $$D=\frac{\sum_{N=1}^{N}\sqrt{4A/\pi }}{N},$$

where *D* is the average value of the equivalent diameters of the solid grains, *A* is the observed area of grain, and *N* is the number of solid grains. Additionally, the mechanical properties of the parts were tested using a material testing machine.

## 3. Results and Discussion

### 3.1. Effects of the Radial Forging Process on the Macroscopic Deformation and Microstructure

**Figure 6:**
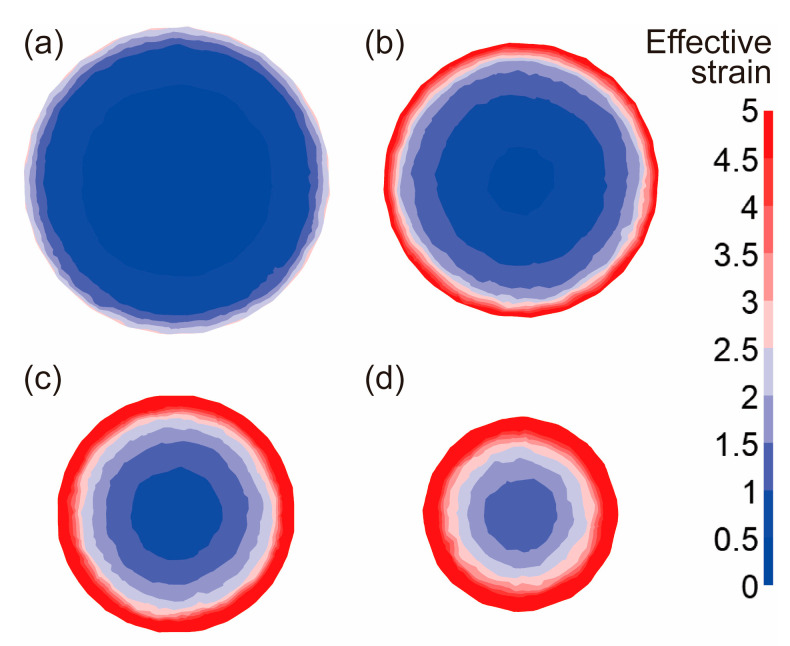
Effective strain distribution of sections with different diameters after forging: (**a**) the area reduction rate of 19%, (**b**) the area reduction rate of 36%, (**c**) the area reduction rate of 51%, (**d**) the area reduction rate of 67%.

**Figure 7:**
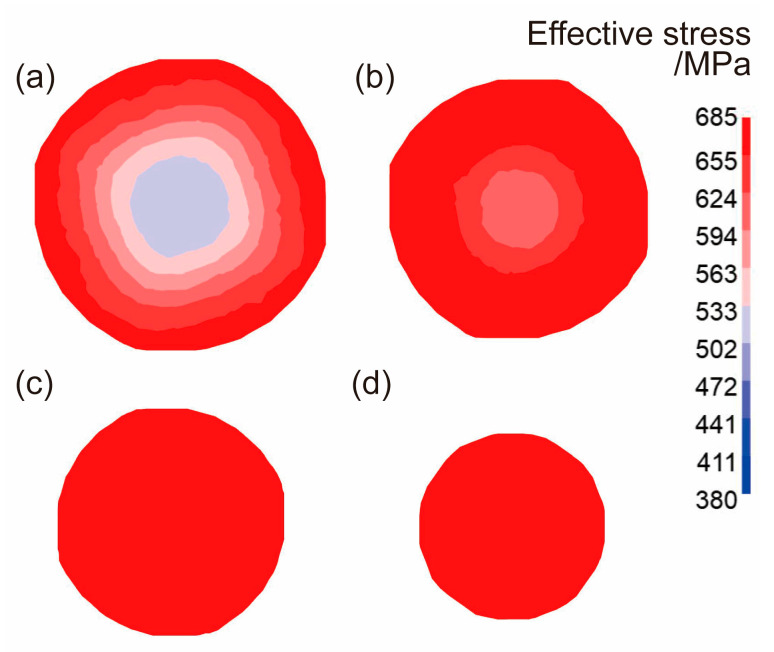
Effective stress distribution of sections with different diameters after forging: (**a**) the area reduction rate of 19%, (**b**) the area reduction rate of 36%, (**c**) the area reduction rate of 51%, (**d**) the area reduction rate of 67%.

The effective strain and effective stress from the cross sections of the radial-forged metal bar with different area reduction rates are shown in Figure 6 and Figure 7. As shown in Figure 6 and Figure 7, when the area reduction rate was 19% after the first forging, the effective strain and effective stress at the edge of the forged bar were approximately 2.5 and 624 MPa, respectively. With an increase in the area reduction rate from 36% to 51%, the effective strain at the edge of the forged bar increased from 2.5 to over 5.0, and the effective strain at the center increased from 0.3 to 0.7, as shown in Figure 6b,c. The effective stress at the edge of the forged bar increased from 624 MPa to 685 MPa, and the effective stress at the center increased from 533 MPa to 655 MPa, as shown in Figure 6b,c. When the area reduction rate was 67% after four forging passes, the effective strain at the edge of the metal bar exceeded 5.0, while the effective strain at the center was above 1.2. Moreover, the effective stress at both the edge and center exceeded 685 MPa, which indicated that the bar material had been better forged. Therefore, with the increase in the area reduction rate, both the effective strain and effective stress of the material also increased, and the maximum effective strain and effective stress were at the edge of the section, and gradually decreased to the center.

**Figure 8:**
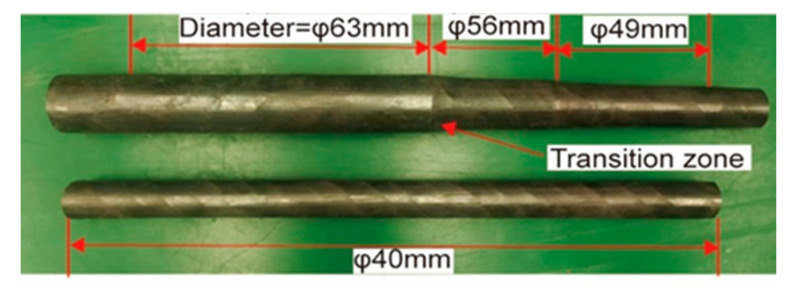
Morphology of the radial-forged AISI 1010 steel bar.

The morphology of the radial-forged AISI 1010 steel bar is shown in Figure 8. As shown in Figure 8, the surface of the radial-forged steel bar with different diameters is smooth, and the shape of this forged bar is good. Moreover, the size error of this forged bar with different diameters is no more than 0.3 mm, which accords with the high precision of the radial forging process.

**Figure 9:**
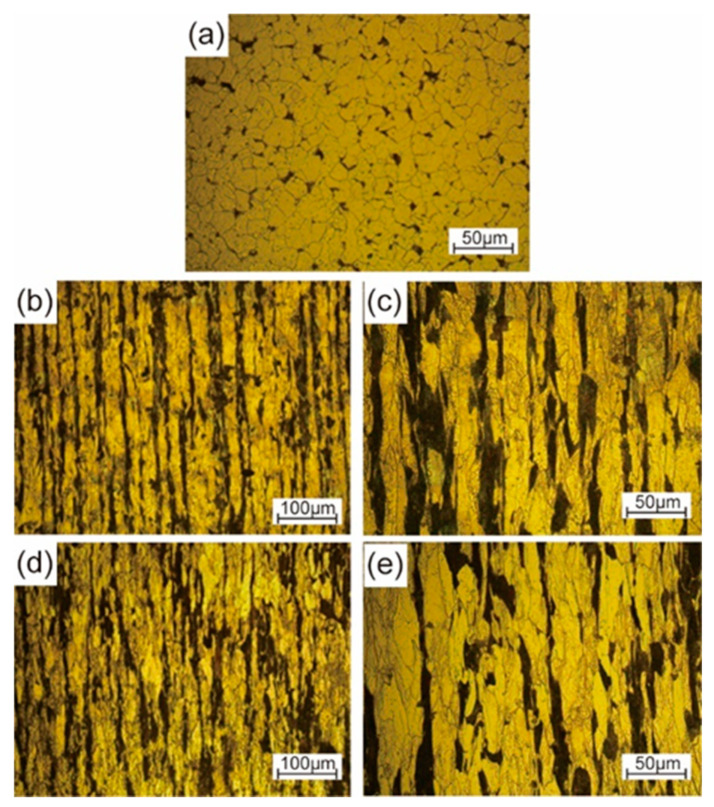
Microstructures of the initial and radial-forged AISI 1010 steel bar: (**a**) initial microstructure, (**b**,**c**) edge position after radial forging, (**d**,**e**) center position after radial forging.

The microstructures of the initial and radial-forged AISI 1010 steel bar are shown in Figure 9. As shown in Figure 9a, the white part, which is the main component, is ferritic, with uniform grain size and clear grain boundaries, while the black bulk is pearlite distributed at the ferritic grain boundaries. Figure 9b–e show the microstructures at the center and edge positions of the radial-forged bar with an area reduction rate of 67%. The grains of the microstructures shown in the Figure 9b–e present a fibrous shape, extending along the radial forging direction, and the radial forging direction is formed due to the radial forging process. The boundaries between the grains are elongated, and the phenomenon of grain breakage occurs, indicating that the radial-forged bar with an area reduction rate of 67% has been completely penetrated from the center to the edge.

During the radial forging process, the outer material of the bar is subjected to radial and circumferential pressure, so the material is forced to flow axially. The larger the diameter, the greater the strain on the material, so the flow of the material along the axial direction is more obvious. Therefore, high strain energy and new grain boundaries are accumulated inside the radial-forged bar because of the radial forging process, which is conducive to the formation of fine and spherical grains during the subsequent isothermal treatment.

### 3.2. Effects of the Isothermal Holding Process on the Microstructures of the Radial-Forged Bar

**Figure 10:**
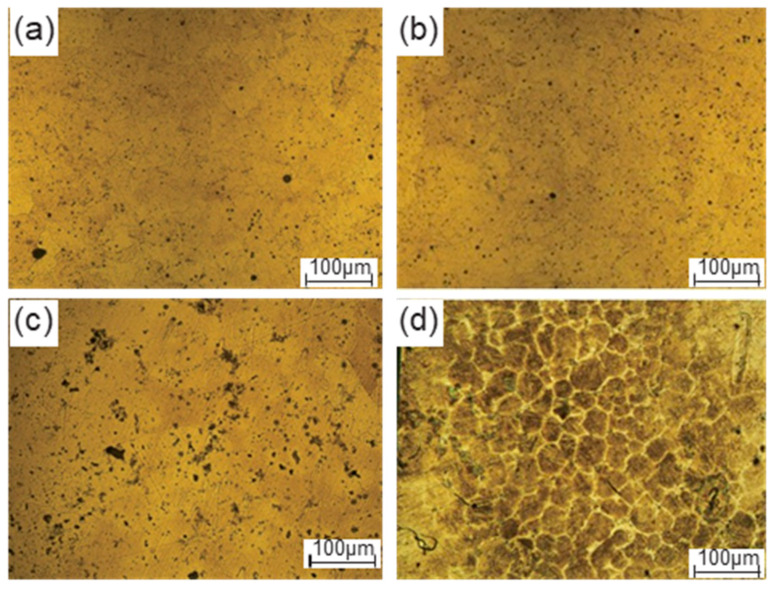
Microstructures of the radial-forged bar with the area reduction rate of 67% after isothermal treatment under different heating temperatures and holding times: (**a**) 1490 °C and 10 min, (**b**) 1490 °C and 15 min, (**c**) 1500 °C and 10 min, and (**d**) 1500 °C and 15 min.

Figure 10 shows the microstructures of radial-forged AISI 1010 steel bars with an area reduction rate of 67% after isothermal treatment under different heating temperatures and holding times. As shown in Figure 10a,b, when the heating temperature was 1490 °C and the holding time was 10 and 15 min, the grain boundary was unclear, and many solid grains were still congregated together, which can be attributed to the low heating temperature. As shown in Figure 10c, when the heating temperature increased to 1500 °C, and the holding time was 10 min, the grains were unclear, while some liquid phase began to appear at the grain boundaries between the grains. When the holding time was extended from 10 min to 15 min, an obvious grain-boundary penetration with the liquid phase was observed, and the grains were fine and globular. The average size of the microstructure for grains is shown in Figure 10d, which was found as 56 μm, indicating that the AISI 1010 steel bar with an area reduction rate of 67% after isothermal treatment at 1500 °C for 15 min was suitable for the semi-solid metal forming. The microstructural evolution mechanism of the semi-solid AISI 1010 steel bar prepared using radial forging and isothermal treatment can be explained as follows: during the radial forging process, the original AISI 1010 steel bar with large ferritic and pearlite can be broken into small ones aligned in the radial forging direction, and large strain energies are stored in the radial-forged billets in the forms of vacancies and dislocation. When the radial-forged billets are isothermally treated at 1500 °C for 15 min, the liquid phase will occur and penetrate the grain boundary, resulting in the fragmentation and refinement of grains, which can be attributed to the high heating temperature and long holding time.

### 3.3. Effects of the Thixotropic Forging Process on the Microstructure and Mechanical Properties

**Figure 11:**
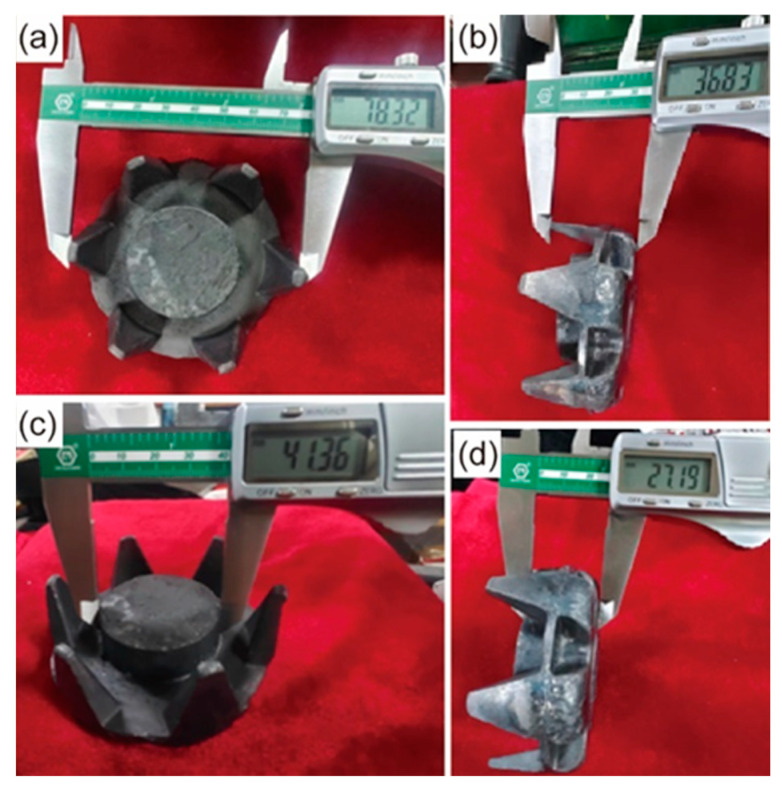
Morphology of the AISI 1010 steel claw pole formed using the semi-solid thixotropic forging forming process: (**a**) outside diameter of the claw pole, (**b**) height of the claw pole, (**c**) outside diameter of the claw pole boss, (**d**) height of the claw pole boss.

The morphology of the AISI 1010 steel claw pole formed using the semi-solid thixotropic forging forming process at the area reduction of 67%, the heating temperature of 1500 °C, the die to preheat temperature of 300 °C, and the die closing speed of 45 mm/s, are shown in Figure 11. The AISI 1010 steel claw pole is fully filled with sharp profiles and flat surfaces, and no obvious macro flaws such as shrinkage and cracks can be seen in Figure 11. Moreover, the main dimensions of claw poles measured with vernier calipers can also be seen in Figure 11. The outside diameter of the claw pole is 78.32 mm, the height of the claw pole is 36.83 mm, and the outside diameter and height of the claw pole boss are 41.36 mm and 27.19 mm, respectively. The comparison between the actual dimension requirements of the claw pole shown in Figure 2, and the semi-solid thixotropic forging results shown in Figure 11, is summarized in Table 3. It can be seen that the actual dimension of the semi-solid thixotropic forging claw pole has reached the required dimensions of the claw pole parts, indicating that the thixotropic forging process of semi-solid AISI 1010 steel billets qualifies under the process parameters listed above.

**Figure 12:**
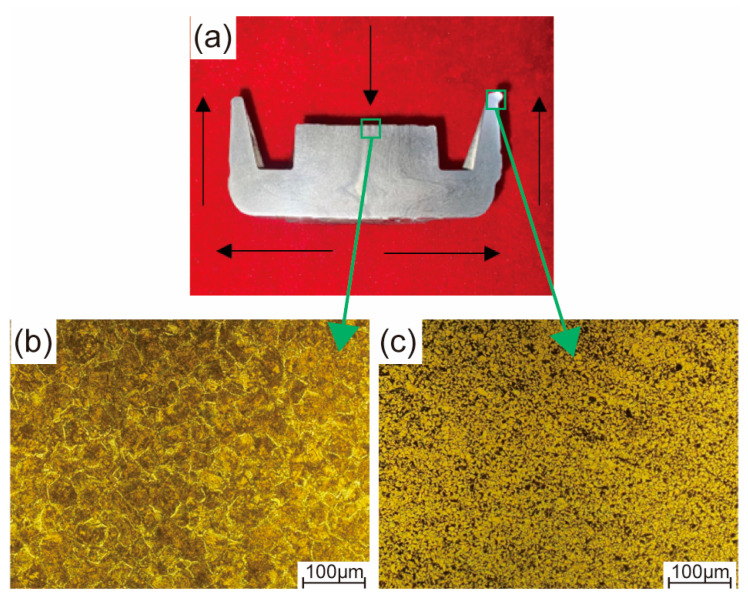
Morphology of the material flow behavior and microstructures of the AISI 1010 steel claw pole formed using the semi-solid thixotropic forging forming process: (**a**) morphology of material flow, (**b**) central position of the claw pole boss, (**c**) top position of the claw pole tooth shape.

The morphology of the material flow behavior and the microstructures of the AISI 1010 steel claw pole formed using the semi-solid thixotropic forging forming process are shown in Figure 12. As shown in Figure 12a, the claw pole was divided in half to investigate the material flow behavior. During the starting stage of the semi-solid thixotropic forging forming of the claw pole, the semi-solid billets were compressed by the top die shown in Figure 5, and expanded along the radial direction. Then, the semi-solid billets flowed upward to form a tooth shape under the restriction of the semi-solid thixotropic forging die side wall, and the claw pole shown in Figure 11 could be formed. It can be seen from Figure 12b that the central-position microstructures of the final claw pole are fine and spherical, and the pearlite is distributed among the ferrite grains, which are typical microstructures of the semi-solid forming parts. Moreover, the liquid phase film between the solid grains is very thin, and the solid grains are congregated together, which can be attributed to the liquid phase being extruded and flowing easily during the semi-solid forming process. Compared with the microstructure at the center position, the microstructure at the top position of the claw pole tooth shape is smaller, and there is a large amount of liquid phase, which further verifies the liquid-phase flowing characteristics during the semi-solid thixotropic forging process.

The mechanical property of the AISI 1010 steel materials processed by the SSTFF process was compared with the annealed AISI 1010 steel (the starting material) used in this work, which is shown in Table 4. It was found that both the yield strength and the tensile strength of the AISI 1010 steel after SSTFF were higher than those without this process. The yield strength of the AISI 1010 steel increased from 210 MPa (before the SSTFF treatment) to 396 MPa (after the SSTFF treatment). The tensile strength was enhanced to 629.4 MPa through the SSTFF treatment. The mechanical property improvement ratios of yield strength and tensile strength were 88.6% and 79.8%, respectively. According to the research of Kim et al. [33], Al-Sahlani et al. [34], Jiang et al. [35], and Bhattacharya et al. [36], the mechanical properties of the material are mainly affected by the shape and size of the microstructure under the condition of the same material compositions, and the improvement of the mechanical properties can be explained through the Hall–Petch formula, as shown in Equation (2) [37], where *σ*_0_ is the intrinsic lattice strength, *k*_y_ is the Hall–Petch coefficient, and *d* is the average size of the grains.


(2) $$\sigma_{y}=\sigma_{0}+k_{y}d^{-1/2}$$

According to the Hall–Petch formula shown in Equation (2), reducing the grain size can increase the yield and tensile strength of the material, because the material flow is hindered by more grain boundaries [38,39,40]. Compared with the average grain size of the annealed AISI 1010 steel microstructures shown in Figure 8a, the average grain sizes of the final claw pole formed using the SSTFF process are finer and denser, as shown in Figure 11b,c. Consequently, the mechanical properties of the AISI 1010 steel in this work were improved by the SSTFF process. Moreover, it can be seen from the results that the SSTFF process can effectively control the shape and size of the microstructure of the material, so as to improve its flow behavior and mechanical properties.

## 4. Conclusions

This paper proposes the SSTF process, including radial forging, isothermal treatment, and thixotropic forging forming, for producing the AISI 1010 steel claw pole. The effect of the area reduction rate on the effective strain and effective stress of the radial-forged metal bar, and the effect of the isothermal holding process parameters, including the heating temperature and holding time, on the macrostructures, the microstructures and the mechanical properties of the claw pole obtained using the thixotropic forging forming process are presented and discussed. Conclusions can be obtained and shown as follows.

(1) When the AISI 1010 low-carbon steel bar was treated using the radial forging process, with an area reduction rate of 67%, the surface of the radial-forged steel bar with different diameters was smooth, and the shape of this forged bar was good. Moreover, the size error of this forged bar with different diameters was no more than 0.3 mm. Therefore, an excellent-quality radial-forged AISI 1010 low-carbon steel bar can be obtained using the radial forging process with different area reduction rates.
(2) When the AISI 1010 low-carbon steel bar was treated using the radial forging process with an area reduction rate of 67%, the microstructures of the AISI 1010 steel bar presented a fibrous shape, extending along the radial forging direction. The boundaries between the grains were elongated, and the phenomenon of grain breakage occurred, indicating that the radial-forged bar with an area reduction rate of 67% had been completely penetrated, from the center to the edge.
(3) Semi-solid AISI 1010 low-carbon steel with an average size of 56 μm can be prepared using the radial forging and isothermal treatment process, and the optimized operation parameters for preparing low-carbon steel semi-solid billets with fine and globular microstructures are an area reduction rate of 67%, an isothermal temperature of 1500 °C, and a duration time of 15 min.
(4) It was found that the low-carbon steel claw pole fabricated using a semi-solid thixotropic forging forming process was fully filled with a sharp profile and a flat surface, the central position microstructures of the final claw pole were fine and spherical, and the pearlite was distributed among the ferrite grains, which were typical microstructures of the semi-solid forming parts. The yield strength and tensile strength from the AISI 1010 steel of the claw pole formed using the SSTFF process increased by 88.6% and 79.8%, respectively, compared to the starting materials.

## Figures and Tables

**Table 1:** Chemical composition of AISI 1010 steel (wt.%).

C	Si	Mn	S	P	Ni	Cr	Cu	Fe
0.10	0.18	0.35	0.035	0.035	0.3	0.15	0.25	Bal.

**Table 2:** Radial forging scheme of the AISI 1010 steel bar.

Forging Step	Diameters after Forging/mm	Area Reduction Rate/%
1	63	19
2	56	36
3	49	51
4	40	67

**Table 3:** Comparison between the actual dimension requirements, and the semi-solid thixotropic forging results.

Main Dimensions	Required Dimensions (mm)	Semi-Solid Thixotropic Forging Formed Dimensions (mm)
Outside diameter of the claw pole	$78_{-0.5}^{+1}$	78.32
Height of the claw pole	36.$5_{-0.5}^{+0.5}$	36.83
Outside diameter of the claw pole boss	$41_{-0.5}^{+0.5}$	41.36
Height of the claw pole boss	$27_{-0.5}^{+0.5}$	27.19

**Table 4:** Mechanical properties of the annealed AISI 1010 steel used in this work, and the AISI 1010 steel of the claw pole formed using the SSTFF process.

Different Materials	The Annealed AISI 1010 Steel (Starting Material in This Work)	The AISI 1010 Steel of the Claw Pole Formed Using the SSTFF Process	Mechanical Properties Improvement Ratio
Yield strength (MPa)	210	396	88.6%
Tensile strength (MPa)	350	629.4	79.8%

## Data Availability

The data presented in this study are available upon request from the corresponding author. The data are not publicly available, due to the project confidentiality requirements.
